# Microsatellite diversity and broad scale geographic structure in a model legume: building a set of nested core collection for studying naturally occurring variation in *Medicago truncatula*

**DOI:** 10.1186/1471-2229-6-28

**Published:** 2006-12-13

**Authors:** Joëlle Ronfort, Thomas Bataillon, Sylvain Santoni, Magalie Delalande, Jacques L David, Jean-Marie Prosperi

**Affiliations:** 1 UMR 1097 « Diversité et Adaptation des Plantes Cultivées », INRA Montpellier, Domaine de Melgueil 34130 Mauguio, France; 2 Department of Genetics and Ecology, Bioinformatics Research Center, University of Aarhus, Høgh-Guldbergs Gade 10, Building 1090, DK-8000 Århus C, Denmark

## Abstract

**Background:**

Exploiting genetic diversity requires previous knowledge of the extent and structure of the variation occurring in a species. Such knowledge can in turn be used to build a core-collection, i.e. a subset of accessions that aim at representing the genetic diversity of this species with a minimum of repetitiveness. We investigate the patterns of genetic diversity and population structure in a collection of 346 inbred lines representing the breadth of naturally occurring diversity in the Legume plant model *Medicago truncatula *using 13 microsatellite loci distributed throughout the genome.

**Results:**

We confirm the uniqueness of all these genotypes and reveal a large amount of genetic diversity and allelic variation within this autogamous species. Spatial genetic correlation was found only for individuals originating from the same population and between neighbouring populations. Using a model-based clustering algorithm, we identified four main genetic clusters in the set of individuals analyzed. This stratification matches broad geographic regions. We also identified a set of "admixed" individuals that do not fit with this population structure scheme.

**Conclusion:**

The stratification inferred is discussed considering potential historical events like expansion, refuge history and admixture between neighbouring groups. Information on the allelic richness and the inferred population structure are used to build a nested core-collection. The set of inbred lines and the core collections are publicly available and will help coordinating efforts for the study of naturally occurring variation in the growing *Medicago truncatula *community.

## Background

In the last decade, model plants have been the subject of rapid advances in genomics, including the completion of the sequence of both *Arabidopsis thaliana *[1] and rice [2,3]. Knowledge gained from these studies and associated technological and methodological progresses create new prospects in a variety of biological fields, including functional and evolutionary genetics. This simultaneously renews our interest in naturally occurring variation. Genomic approaches to the study of natural variation should increase our ability to understand gene function [4], while the availability of sequence data from genome-wide marker loci can provide new insights into the major historical and selective factors shaping the genetic diversity of a species [5,6]. Such new prospects for plant natural variation will only be met if carefully constructed samples of genotypes are used for characterizing patterns of naturally occurring variation. This revives interest in analyzing material currently available in germplasm collections. Nearly a century has been spent collecting and preserving genetic diversity in plants, resulting in worldwide collections, currently stored in international networks of seed banks [7]. The large size of most of these collections however restricts the characterization of the material available, and hinders their successful use.

To "unlock" the genetic potential of these large collections, a general proposal is to construct smaller "core-collections", i.e. sub-samples of accessions chosen to represent the bulk of the genetic diversity contained in the larger collection. Different sampling strategies have been proposed. Genetic markers can be used to characterize the genetic variation present in the collection. Such knowledge can lead to significant gains in the number of alleles retained in a sample compared to random sampling. First, the observed DNA profiles can be used to determine the genetic uniqueness of each accession relative to all others and to reduce redundancies in the collection. Second, analysis of single or multilocus genotypes allows inference of genetic ancestry among accessions. A model-based clustering algorithm that identifies groups with distinct allele frequencies [8,9] can then be used to stratify the collection into a series of groups, even without prior information about the sampling locations of individuals. Subsequently, core collections can be established by stratified sampling from the different groups [10]. An alternative way to use molecular information for sampling is to use marker gene data as a way to score the allelic richness of a sample. This approach, known as the Maximization strategy, or hereafter *M *strategy, chooses the specific combination of accessions that maximizes the total allelic richness at available marker loci [11]. Simulations showed that the efficiency of this method is expected to increase with increasing selfing rate and reduced gene flow in the studied species [12]. These predictions were recently validated using data from *Arabidopsis thaliana *and, although the gain in diversity was rather modest [13], the *M *strategy was used to build a set of nested core collections that can be used as a "gateway" to explore naturally occurring diversity in this species.

*Medicago truncatula *recently emerged as a model species for the analysis of development during microbial interactions and other aspects of legume genetics and genomics [14]. Key attributes of this species include its small, diploid genome (~5*10^8 ^bp), its self-fertile nature and its prolific seed production. Researchers are developing tools and methods for molecular and genetic analysis [15], and an international consortium is currently sequencing the "gene space" of *Medicago truncatula *[16]. Through the use of forward genetic strategies, these resources have already enabled the identification of a number of new genes that participate in the recognition of microbial and mycchorizal symbionts [17-21]. Until now, most studies in *Medicago truncatula *have focused on either a single reference individual or a limited number of populations [22,23], with the exception of one recent study of 192 accessions [24]. In the present paper, we use a set of 13 microsatellite markers to survey the genetic diversity occurring in a sample consisting of 346 inbred lines spanning the bulk of the diversity that has been collected throughout the species range to date (Table 1). First, we aimed to quantify the neutral genetic diversity available in this collection and to test for the presence of an underlying genetic structure in this broad sample. In a second step, results from these analyses are used to generate a set of nested core-collections for *Medicago truncatula*. This subset of inbred lines represents the bulk of the diversity segregating in *M. truncatula *collected so far and is publicly available. These core collections will help organize and coordinate current efforts in the *Medicago truncatula *community to study patterns of variation at both the phenotypic and the molecular levels.

## Results

### Microsatellite diversity

A summary of the microsatellite polymorphism uncovered is presented in Table 3. The average number of alleles per locus was *N*_*A *_= 20.7 but dropped to 5.8 when removing rare alleles (i.e. with a frequency lower than 0.05). Averaged over loci, the gene diversity was *H*_*E *_= 0.75 with large variation among loci (SD = 0.30; Table 3). Rare heterozygote genotypes were found for each of the studied loci (*H*_O _> 0) but were dispersed across individuals [see Additional file 5]. Among the 78 pairs of loci, 29 were found to be in significant linkage disequilibrium (Table 4). These pairs involved all the loci studied except the two less variable ones (MTIC126 and MTIC143) which were in linkage equilibrium with all the other loci. Locus MTPG85C contributed to 9 of the 29 statistically associated pairs of loci. Combining data from the 13 microsatellite loci, we found 346 different multilocus profiles, thereby confirming the genetic uniqueness of each inbred line in our sample.

### Genetic structure of the collection

Spatial autocorrelation analyses conducted over the whole sample revealed a weak signal of isolation by distance. Positive and significant associations between kinship coefficients and geographic proximities were found only for individuals originating from the same location (collected in the same natural population) and between neighbouring populations. Similar analyses were conducted within two well-represented geographic regions: Spain and Algeria. Kinship coefficients were of the same order of magnitude and we did not find any clear evidence for isolation by distance even at this restricted spatial scale (data not shown).

We tested for the existence of a broad genetic stratification in our sample, consisting in *K *underlying genetically diverged groups (hereafter clusters), using microsatellite data and the model based clustering algorithm implemented in the software *Structure *(see methods section for details). To do so, we built a subsample of 266 accessions representing each sampled location by a single (randomly chosen) inbred line. Despite its smaller size compared to the initial sample, this subset exhibited similar levels of genetic diversity: mean *N*_A _= 19.8, mean *H*_E _= 0.75 (SD = 0.30). The pattern of linkage disequilibrium among loci was also conserved, with 22 out of 66 possible pairs of loci showing statistically significant associations (compared to 29 out of 78 pairs in the whole data set). Inspecting the gain in likelihood of the data (*n *= 266) when modeling the data using an increasing number of underlying clusters (from *K *= 1 to *K *= 8) yielded clear support for the existence of a genetic structure in our sample. Consistent estimates of Log Likelihood of the data across independent runs were obtained, with a slight variation between runs for *K *= 5. Posterior probabilities of the data always increased with increasing subdivision; the highest gain occurred between *K *= 1 and *K *= 2 and we found an inconspicuous plateau between *K *= 4 and *K *= 5 [see Additional file 2]. Independent runs produced highly consistent results for all *K *values except for *K *= 3 and *K *= 8 which yielded more than 30 inbred lines displaying inconsistent assignation between runs (this was found in less than 10 cases for all the other investigated *K *values).

**Figure 1:**
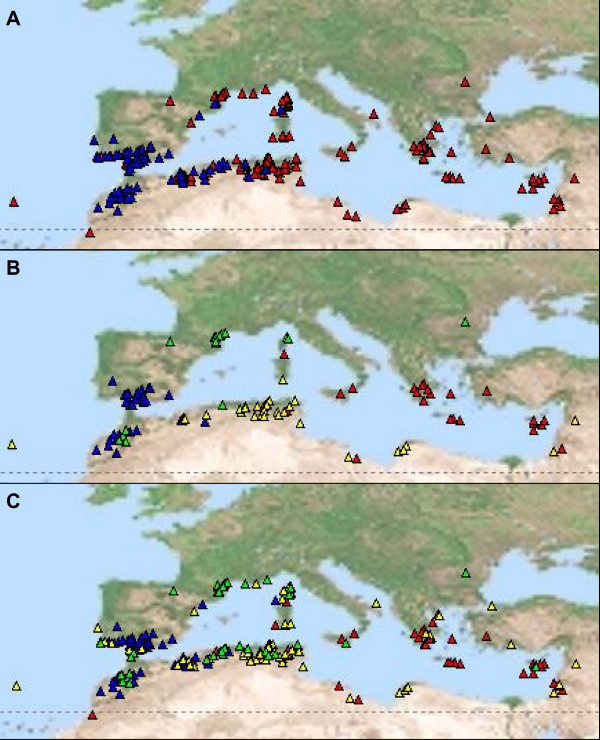
**Geographical distribution of individuals comprising the groups inferred using microsatellite polymorphism**. The stratification of the collection obtained assuming *K *= 2 (A), or *K *= 4, plotting only accessions assigned with a high membership into a group, *p *> 0.6, (B), or plotting all the individuals (C). We used the output yielding the highest posterior probability to assign each genotype to a group.

At *K *= 2, the inferred structure was totally stable across runs and geographically consistent, isolating a set of 78 accessions originating from the South of Spain and from Morocco (Figure 1a). This stratification accounted for 6.5% (*F*_ST _= 0.065, *p *< 0.001) of the total genetic variance. At *K *= 3, independent runs were less consistent, splitting roughly the largest cluster obtained at *K *= 2 in two groups, one being mainly represented by Algerian accessions. At *K *= 4, approximately 65% of the accessions showed a proportion of membership higher than 0.6 into a group. Plotting these "well-assigned" accessions on a geographic map showed that the clusters corresponded largely to major geographic regions (Figure 1b). One cluster corresponded to the cluster already observed at *K *= 2, i.e. grouping populations from Spain and Morocco (referred as cluster 2 hereafter). Another cluster consisted largely of individuals collected in the South of France (hereafter cluster 4) while the two last groups distinguished the North-Eastern (cluster 1) and the South-Eastern of the Mediterranean Basin (cluster 3, see figure 1b). Plotting accessions with low partial membership (maximal proportion of membership into a group lower than 0.6) blurred this geographic stratification (Figure 1c).

For *K *> 4, each increase in *K *split one of the clusters obtained at previous *K *values. However, although the posterior probability of the data slightly increased with increasing *K *value, the mean proportion of membership into the inferred clusters jointly decreased, with more and more accessions showing a maximum membership into a group lower than 0.6. This result combined with the plateau observed at *K *= 4 lead us to consider that the stratification observed at *K *= 4 was probably the most genuine one. The highest probability run observed at *K *= 4 was consequently used to define the different clusters. Finally, re-running the clustering procedure on the 346 accessions and using the predefined population structure as 'prior information', we assigned the 80 supplementary accessions into these four clusters [see Additional file 3]. Although most of these accessions showed partial membership in multiple clusters, 50 out of the 80 accessions were assigned to the same cluster as other accessions originating from the same population. After this last step, the four clusters inferred were composed of respectively 83, 99, 114 and 50 accessions. The cultivars Cyprus, Caliph and Paraggio were assigned to the North-Eastern group (in red Figure 1c). Cultivars Borung, Mogul and Sephi were assigned to the South-Eastern group (in yellow, Figure 1c) while Jemalong was assigned to the Spanish-Moroccan group (in blue, Figure 1c).

Analyzing patterns of genetic diversity within this stratification showed that the levels of genetic diversity is relatively homogeneous across clusters, each group exhibiting approximately the same level of genetic variation, as measured by *H*_E _or *N*_A _(Table 4). Compared to the whole sample, the four clusters showed lower proportions of significant pairwise linkage disequilibrium, with a maximum of 5 pairs of loci in LD for the smaller group (i.e. cluster 4 mainly composed of French accessions). Between clusters pairwise *F*_ST _estimates varied between 0.05 and 0.10, the largest *F*_ST _values were found in all pairwise comparisons involving cluster 2 (i.e. the Spain-Morocco group); the lowest values being observed between the Northern and the Southern clusters (Table 5).

### Sampling of core-collections

**Figure 2:**
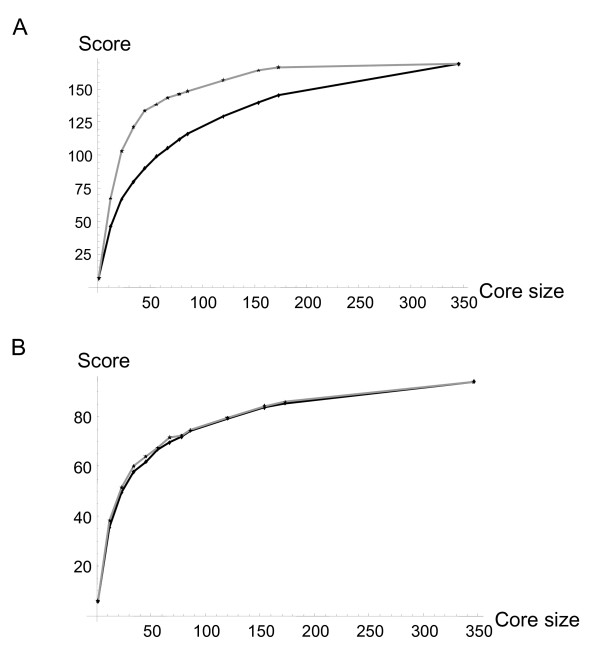
**Comparison of the efficiency of random and "M" sampling strategies**. (A) The score, calculated as the number of alleles captured in each core collection summed over 6 loci chosen as marker loci, of a pure random strategy (black) and the M strategy (grey) are compared for various Core (collection) sizes. For each core size, the score of each method reported here is an average over 30 independent core collections. (B) The score, calculated as the number of alleles captured in each core collection summed over 7 loci chosen as target loci, of a pure random strategy (black) and the M strategy (grey) are compared for various Core (collection) sizes. For each core size, the score of each method reported here is an average over 30 independent sampled core collections. See methods for details about the implementation of each sampling strategy.

**Figure 3:**
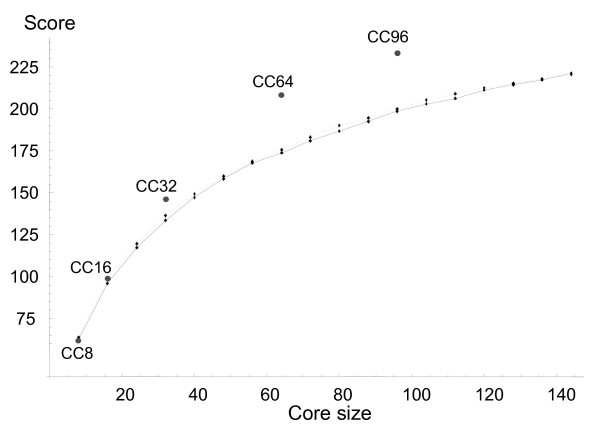
**Comparison of the efficiency of random and stratified sampling strategies**. The scores, the number of alleles captured in each core collection summed over the 13 loci used in this study, of a pure random strategy (black) and a stratified random strategy (grey) are compared for various Core (collection) sizes. For each core size, the score of each method reported here is an average over a hundred independent core collections. The score of the set nested core collections retained to represent the diversity of our *Medicago truncatula *collection (CC8 to CC96) are represented by grey circles.

We first studied the performance of two marker based strategies; the *M *and the *H *strategies (see Methods for details) to build core collections by sampling our total data set of 346 genetically unique inbred lines. The performance of these two sampling strategies for assembling core collection was studied over a range of putative core collection (sample) sizes. For each sample size, the performance of each strategy (*M, H*) was compared to a pure random strategy by comparing the average score of 30–100 core collections sampled independently. When evaluating the *M *strategy, we used 6 loci as markers to implement the *M *strategy and we used the remaining 7 loci as targets to cross validate the efficacy of the *M *strategy. Such procedure allowed us to test whether the *M *strategy can assemble core collection displaying high allelic diversity not only at the set of markers used to implement it but also throughout the genome. Not surprisingly the score of core collections built using the *M *strategy was much better than pure random sampling when considering marker loci only (Figure 2a). However, when considering a set of 7 loci not used by the *M *strategy, the score of core collections built by the *M *strategy was only slightly better than the score of core collections sampled purely at random (Figure 2b). This finding suggests that relatively modest gains in allelic diversity are to be expected when building core collection using the *M *strategy relative to a pure random sampling of the collection. Similarly we compared the score of core collections built using the *H *strategy with purely random core collection of the same size. The average score of the *H *strategy was consistently better than a pure random sampling but the difference in score was nevertheless very small (Figure 3).

A set of nested core collections were assembled to represent the bulk of the diversity contained in our collection of 346 genotypes. These core collections were composed of 8, 16, 32, 64, 96 individuals and are denoted as CC8, CC16, ..., CC96 [see Additional file 4]. Given the performances of the *M *and the *H *strategies, we finally used a "hybrid/mixed" strategy to assemble these collections. For each core size, we gave equal weight to each of the four clusters uncovered in the structure analysis. Within each cluster, we used the *M *strategy to build a core collection of *c *individuals. Given that a number of important inbred lines (used as parents for mapping populations) were found in all four clusters inferred when stratifying our collection in *K *= 4 groups, we chose to retain these 8 genotypes as our starting CC8 collection. For all subsequent core collections (CC16, CC32, etc.), the *M *strategy was implemented within each of the four groups and constrained to include the individuals comprising the "previous" core collections. This yielded a set of nested core collections whose detailed composition is provided to help design further studies [see Additional file 4]. The allelic richness of each core collection is displayed Figure 3.

## Discussion

### Microsatellite variation in *Medicago truncatula*

Surveying a worldwide collection of individuals for a set of 13 microsatellite loci distributed throughout the genome, we revealed a large amount of genetic variability in *Medicago truncatula*. This result was expected considering the sampling scale and the class of markers we used. Microsatellite markers are known to display high mutation rates, and are thus expected to reveal fairly high amounts of polymorphisms especially when used at the species level [25]. We found however a large variation among loci for both the gene diversity (*H*_e_) and the allelic numbers, with two loci exhibiting less than 6 alleles over the whole sample. Comparisons with other annual selfing plant species are not straightforward as we used a large sample size spanning a wide geographic range. A survey of *Arabidopsis thaliana *conducted at a broad geographic scale over 71 individuals reported a similar range of values for *H*_E _(9 loci, 0.79–0.96) [26]. This range is similar to the range found here when excluding the three loci exhibiting markedly reduced levels of polymorphism (MTIC126, MTIC243 and MAL367466). MTIC126 and MTIC243 were based on EST sequences. This could explain their lower polymorphism compared to other loci located in less constrained intergenic regions

Multilocus level analyses revealed no redundancy within the collection, so we used all the individuals for further analyses. The sample we used spans different spatial scales: the population level, different geographical regions and the whole species. A hierarchical analysis would thus appear appropriate to determine how genetic diversity is organized within and between populations. Such an analysis was however not suitable because the within population sampling scheme was not random: sampling was based on prior information on allozyme genotypes and was performed to maximize the within population diversity. In *Medicago truncatula*, the fine-scale population structure has already been documented. From these studies, we know that within population diversity in *Medicago truncatula *is highly variable among populations (mean *H*_E _ranging from 0.10 to 0.24 using RAPD markers [27] and reaching values as high as *H*_E _= 0.48 when using microsatellite markers [23]).

Previous hierarchical population genetic analyses also revealed that pairs of populations are highly differentiated even at a small spatial scale (*F*_ST _values as high as 0.39 are observed between populations located 30 meters apart, [23]). In accordance with these results, the present study was able to demonstrate a spatial genetic correlation only for individuals originating from the same population or from very adjacent populations. The occurrence of spatial correlation over short distances can be interpreted as the result of current low distance migration/drift processes. For a selfing species, most dispersion should occur through seed dispersal. In *Medicago truncatula*, short distance seed dispersal can occur (JR, pers. observation) but the fruits bear spikes which facilitate dispersal by animals (grazing mammals) potentially over longer distances. Moreover, *M. truncatula *is often confined to marginal agricultural habitats, and thus undergo rapid population turnover. This type of so called metapopulation dynamics is expected to increase genetic drift at the population level. These factors are expected to generate highly differentiated populations with neighbouring locations showing as much differentiation as much more distant one as we observed here. This configuration, combined with the sampling scheme adopted in our survey, could also explain the low level of linkage disequilibrium observed in the whole sample [28,29]. Ostrowski and collaborators [26] also recently reported a similar finding in a broad sample of *Arabidopsis thaliana *accessions.

### Inferred population structure in *Medicago truncatula*

Although we could not find isolation by distance over large geographic distances, the model-based clustering algorithm we used revealed patterns of population structure that were roughly consistent with the geographic origin of the accessions. This consistency was striking when considering accessions with a high proportion of membership in one of the inferred clusters. Accessions with partial membership (highest membership <0.6) shuffled the tight correspondence between genetic ancestry and regional affiliation. These accessions represented roughly 30% of the sample, and probably signed recent events of gene-flow between neighbouring groups or a mixture of large distance dispersal and recombination events. The population structure inferred at *K *= 4 was retained as the most consistent stratification because high proportions of individuals with partial membership (<0.6) were found for higher *K *values [see Additional file 3]. This stratification accounted for 8% of the microsatellite genetic variation observed over the whole sample, and, as expected from the clustering algorithm we used, the within cluster levels of linkage disequilibrium were low compared to the whole sample. We note that caution must be exerted when interpreting the biological significance of this stratification. In particular the number of inferred clusters should not be taken at face value as it reflects the underlying true genetic structure, which may be discrete or continuous, its sampling and the amount of marker information available for inference [30]. With that in mind, we discuss below the most salient features of the structure we uncovered.

The cluster showing the highest geographic consistency corresponded to a restricted geographic area: the Iberian Peninsula and Morocco. Interestingly, this cluster was already identified at *K *= 2 and subsequently displayed the highest level of differentiation with the three other clusters (at *K *= 4). This cluster was also clearly differentiated from both France and Algeria. Together, these results provide compelling support for the previous hypothesis of a refuge during glacial cycles in this region [31]. Iberia has been shown to be one of the three main European refugia for most plant species along with Italy and the Balkans [32-34]. For the remaining accessions, the stratification uncovered at K = 4 clearly distinguished the North and the South of the Mediterranean basin. This result suggests a colonization of the Mediterranean via two routes from an initial area located around the Middle East. The low level of differentiation observed between these two groups (Northern Mediterranean vs. Southern Mediterranean) however can also be interpreted as the result of a recent expansion of the species around the Mediterranean.

### Performance of core collection sampling strategies

When benchmarking the *H *and *M *strategies against a pure random strategy, we found very little gain when scoring genetic diversity at loci not involved in the sampling (Figure 2b and Figure 3). This suggests that the *H *and *M *strategies are not very likely to perform better than a pure random sampling. This finding may be surprising given that the mating system of *M. truncatula *(high levels of selfing) and the patterns of regional subdivision uncovered in our sample are factors that *a priori *favor marker assisted sampling strategies over pure random sampling to capture efficiently variation [12]. One particular property of our sample may however explain this finding. The set of inbred lines to which we applied these sampling strategies were highly non redundant to start with. In fact, for all populations coming from the INRA collection that make up 2/3 of our sample, the strategy used to extract inbred lines from populations did take into account the amount of polymorphism detected within populations. This strategy ensured for instance that populations which displayed relatively higher levels of variation were represented by more inbred lines (up to 8 inbred lines were extracted per population) than populations found to be monomorphic. Thus the initial absence of redundancy in our sample of 346 inbred lines (all being genetically unique) together with overall low levels of linkage disequilibrium may leave little room for the optimized sampling of variation and may explain the poor gains of marker-assisted sampling strategies (*H *and *M*) over pure random sampling. Another possibility is that more markers or markers with lower mutation rates may be needed to efficiently "tag" the genome. McKhann et al [13], using only 10 gene anchored fragments (of about 600 pb) and SNP variation present within those fragments to guide sampling observed some modest gain (~10%) of the *M *strategy over a pure random strategy.

## Conclusion

We have extracted 346 genetically unique inbred lines from a large set of sampling locations representing the extent of natural variation collected to date throughout the species range of *Medicago truncatula*. These inbred lines, a publicly available resource for the *Medicago *community, were genotyped at 13 microsatellite loci. We used patterns of microsatellite diversity to uncover the presence of a broad scale genetic structure in *M. truncatula*. This stratification, the presence of 4 genetically diverged groups which were inferred solely on the basis of marker data, was found *a posteriori *to be consistent with the geographical origin of genotypes. Pattern of microsatellite diversity and genetic structure in *Medicago truncatula *were used to obtain some insight into the demographic history of this species and to build a set of nested core collection representing the breadth of naturally occurring genetic variation. These, nested core collections can be used as a standardized panel for coordinating efforts aimed at in depth characterization of phenotypic variation and efficient SNPs discovery in *Medicago truncatula*. As such these nested core collections will be a pivotal resource for functional and evolutionary genomics studies in the growing *Medicago truncatula *community. Future work will document the extent of genetic divergence existing between this genepool, the related sub species *M. truncatula ssp tricycla *and the sister species *Medicago littoralis*. This will yield a clearer picture of the phylogenetic relationships between these taxa and will allow identifying a set of accessions complementing our nested core collections.

## Methods

### Plant material

*Medicago truncatula *is native to the Mediterranean and has become naturalized in other regions of the world following European migrations. In Australia, it is used as a forage crop and soil improver. *M. truncatula *has been split into three subspecies mainly on the basis of pod characteristics: ssp *truncatula*, ssp *tricycla *and ssp *longeaculata*. Previous molecular analyses demonstrated that individuals from the subspecies *tricycla *are genetically differentiated from the two other subspecies [24,35]. To avoid genetic stratification at the sub-species genetic subdivision conflicting with patterns of regional subdivision (see below), we did not include *M truncatula *ssp *tricycla *accessions in our sample.

We used the French *Medicago truncatula *collection maintained in Montpellier. For *Medicago truncatula sensu lato *(i.e. including *M. truncatula *ssp *tricycla*), this collection consists of a set of 350 natural populations collected by the laboratory in different countries around the Mediterranean basin (France, Spain, Portugal, Algeria, Greece, Crete) and a set of 231 introductions provided by the Australian Medicago Genetic Resources Center (AMGRC) maintained by the South Australian Research and Development Institute (SARDI, Adelaide, Australia). The set of natural populations collected by our laboratory was previously screened using isoenzymatic markers: for each population, 11 individuals were genotyped (J-M. Prosperi, unpublished data). These data were used to sample different individuals from local populations, ensuring that these individuals were genetically distinct and not mere sibs. Following this procedure, 1 to 8 different individuals were sampled in each population and selfed for two successive generations to obtain inbred lines. For the set of populations obtained from the AMGRC, a single individual was randomly chosen for each accession number and selfed for two consecutive generations. For the present study, 338 inbred lines were chosen in this material in order to span the range of eco-geographical distribution of *Medicago truncatula *(excluding ssp *tricycla*) and to represent different units of spatial structure: population, regions and the whole species. We only consider inbred lines originating from accessions for which we knew at least the precise geographical location. A set of 8 inbred lines representing cultivated populations (hereafter cultivars) was added to the sample, bringing the sample size to 346 (Table 1). A detailed list of the 346 accessions and eco-geographical data are available [see Additional file 1]. Taxonomically speaking, all these 346 accessions come from two closely related subspecies: *M. truncatula *ssp *longeaculata *and *M. truncatula ssp truncatula*.

### Microsatellite genotyping

DNA was extracted from 100 mg of frozen leaves according to the DNeasy Plant Mini Kit (Qiagen). Thirteen microsatellite loci were used for genotyping (Table 2). Six of them have been described previously (AY294632, AY294633, AY294635, AY294640, AY294642, AF274878) [35]. Seven new loci were developed for this study from published sequences (Huguet, pers. com. and Santoni, unpublished results). To do so, *Medicago truncatula *sequences were retrieved from the GenBank database and searched for SSR motifs (dinucleotide and trinucleotide) using the FINDPATTERNS program of the GCG Wisconsin Package (Genetics Computer Group).

Amplification reactions were performed in a final volume of 20 μl in the presence of 20 ng of template DNA, 4 pmol of the reverse primer and 1 pmol of the forward primer, 0.2 mM of each deoxynucleotide, 2 mM MgCl2, and 0.5 unit Taq polymerase (Sigma). The forward primer was 5'-labeled with one of the three fluorophores (6FAM, NED or HEX). PCR was carried out using a PTC 100 thermocycler (MJ Research). After 5 min at 94°, 30 cycles were performed with 30 s at 94°C, 30 s at either 50, 55 or 60°C (depending on the locus), and 30 s at 72°C, followed by final extension step of 5 min at 72°C. Amplified products were detected on an ABI prism 3100 Genetic Analyser. Samples were prepared by adding 3 μl of diluted PCR products to 6.875 μl formamide and 0.125 μl GenSize 400 HD Rox. Analyses were performed using the GeneScan 3.1 and Genotyper 2.5 softwares (Applied Biosystems).

Analyses of microsatellite diversity were conducted at the locus level. For each locus, we estimated the number of alleles (*N*_A_), allelic frequencies, the observed (*H*_O_) and expected (Nei's diversity, hereafter *H*_E_) heterozygosities using Genetix version 4.04 [36]. Statistical associations among loci (linkage disequilibrium) were tested through Fisher's exact tests using the package GENEPOP version 3.3 [37], and corrected for multiple testing was done using a sequential Bonferroni procedure. To determine the genetic uniqueness of each accession and to quantify redundancy, the multilocus DNA profile of all the inbred lines were compared by pairs.

### Inference of population structure

Spatial autocorrelation analyses were conducted using SPAGeDI (version 1.1) [38] which calculates conditional kinship coefficients for all pairs of individuals at various geographical distances (each inbred line being assigned the geographical coordinate of the original site of collection). To test whether the degree of relatedness between individuals depends on the geographical distance, we used a procedure implemented in SPAGeDI which performs permutations of individual locations to determine the relationship between genetic ancestry and spatial distance expected under the null hypothesis of no isolation by distance.

To infer population structure in this broad sample, we used a model-based clustering algorithm implemented in the computer program Structure version 2 [8,9]. This algorithm uses multilocus genotype to identify a predetermined number (*K*) of clusters that have distinctive allele frequencies and assigns portions of individual genomes to these clusters. Since *Medicago truncatula *is an autogamous species (and thus largely homozygous), we used a haploid setting. In the sample scored for microsatellite markers, some populations were represented by more than one inbred line. Because this sampling scheme appears inappropriate regarding the modeling assumptions of Structure [30] we investigated the population structure of our sample in two successive steps. In a first step, we built a new sample drawing at random a single individual in each local population (which resulted in a sub-sample of 266 individuals, see result section). Following [9], we used the "admixture model" assuming "no correlation among allele frequencies". For each run, we used a burn-in period of 10^5 ^MCMC iterations and then 10^6 ^iterations for estimating the parameters. Five runs were considered for each *K *value (*K *is the number of clusters to be inferred), for *K *ranging from 1 to 8 [see Additional file 2]. For each run output, individual accessions were assigned into a group according to their highest proportion of membership into this group.

The clusters thus defined were compared among runs for each *K *value. The choice of the appropriate *K *value was conducted as recommended by the authors of Structure[8]. For each *K *value, we first verified that individual proportions of membership into a group were different from 1/*K*. We also evaluated the stability of the groups' composition between different runs at each *K *value. When these conditions were met, we analysed the behaviour of the Log Likelihood of the data as a function of K, looking for either a maximum value or a more or less plateau for increasing *K*. The lower value of *K *showing such behaviour was considered as representative of the most genuine stratification. For this *K *value, we chose the run yielding the highest probability of the data and assigned each individual into the cluster in which it has the highest fraction of its genome. This stratification was used in a second step to assign the remaining individuals (those removed from the first sample) to the *K *inferred groups. To do this, we ran Structure on the whole data set (*n *= 346), using the 'prior information model'. Here, the clustering inferred in the previous stage was used as "prior information"; Structure was then able to assign the remaining individuals to the *K *previously defined groups, on the basis of their microsatellite genotype.

To measure the fraction of the observed genetic variation explained by the inferred clusters, we used the parameter *F*_ST_, widely used to estimate the between populations component of variation [39] in population structure analyses. Pairwise *F*_ST _were computed on the inferred stratification, using the software GENETIX. The statistical significance of these *F*_ST _values was tested through 1000 permutations of individuals across groups. Statistical associations between loci were tested within each cluster through Fisher's exact tests using *Genepop *version 3.3 [37].

### Core collection sampling

We studied the performance of two different sampling methods using marker information to generate core collections: the Maximization, hereafter *M *strategy [11] and a stratified sampling, hereafter *H*, strategy (see below). Both the *M *and the *H *strategies use information brought by *m *marker loci. We define the score of a core collection as the number of alleles summed over the *m *microsatellite loci. The *M *strategy consists in searching through the (vast) space of all potential core collections of a given size (in number of individuals) that can be formed from the collection of 346 genotypes included in that study and retains the core collection(s) that exhibit the highest score. To implement the *M *strategy, we used the heuristic algorithm [40] implemented in the publicly available MSTRAT software, version 4.1 [41].

As an alternative way of using molecular information, the *H *strategy assumes that the collection has been previously clustered in *K *groups (see section above for the description of the clustering method we used). Each group *i *(i = 1, .., *K*) is characterized by its mean genetic diversity *q*_i_. Various parameters can be used to characterize the genetic diversity of a group, but following Schoen and Brown (1993) we used Nei's diversity (*H*_*E*_). Building a core collection of size *c *(in number of individuals) under the *H *strategy consists in sampling at random *c*_i _= *c q*_i_/*Q *individuals (i = 1, ..., *K*), where *Q *= ∑_i _*q*_i _(i = 1, ..., *K*), in each group. To implement the *H *strategy we partitioned the entire collection in *K *= 4 groups composed respectively of 83, 99, 114 and 50 individuals. This partition corresponds to the one proposed by the software Structure based on the information brought by the 13 microsatellite loci. Despite some variation in group size, all four groups displayed very similar levels of genetic diversity as measured by either the mean number of alleles per loci or the mean diversity of each loci (Table 3). We therefore sampled an equal number of individuals in each groups, i.e. we used *q*_i _= 1/4 (i = 1, ..., 4), to implement the *H *strategy. The *H *strategy and all simulations described below were implemented using the language *Mathematica *version 4.0 [42] (a *Mathematica *notebook is available upon request). The sampling efficacy of both the *M *and the *H *strategies were assessed by comparing the score of core collections built of increasing sizes (in number of individuals) and we used the score of core collections sampled randomly throughout the same collection as a benchmark for these marker-based sampling strategies.

## Authors' contributions

JR and TB participated in the design of the study, analyzed the data and wrote the manuscript. SS participated in the design of the study, developed the new microsatellite markers reported in that study, was responsible for obtaining the molecular data, and participated in the drafting of the manuscript. JMP conceived of the study, and together with MD participated in its design and coordination, and helped to draft the manuscript. All authors read and approved the final manuscript.

## Supplementary Material

Additional File 5 Table S4 Microsatellite genotyping data (*n *= 346). Click here for file

Additional File 2 **Figure S1 (Log) Likelihood of the data (*n *= 266) as a function of *K *(the number of groups used to stratify the sample)**. For each *K *value, 5 independent runs were considered. Click here for file

Additional File 3 Table S2: Composition of groups obtained for different values of *K *(*n *= 266). Click here for file

Additional File 4 Table S3 Composition of the set of nested core-collections assembled to represent naturally occurring variation in *Medicago truncatula *ssp *truncatula*. Click here for file

Additional File 1 Table S1 list of the 346 accessions (inbred lines) analysed in the present study and associated geographical data. Click here for file

## Figures and Tables

**Table 1:** Geographical origin of the 346 *M truncatula *inbred lines used

Geographical origin	Number of lines from SARDI collection	Number of lines (# populations) from the INRA-Montpellier collection
***North Africa***		
Algeria	18	80 (43)
Tunisia	21	-
Morocco	20	-
Libya	9	-
Madeira	1	-
***South-Western Europe***		
Spain	2	58 (37)
Portugal	6	9 (5)
France	3	41 (26)^1^
***Southern Europe***		
Italy	11	-
Greece	9	20 (15)^2^
Turkey	3	-
***Middle East***		
Israel	5	-
Syria	2	-
Jordan	2	-
Cyprus	12	-
***Other***		
Australia	3	-
South Africa	2	-
Romania	1	-
***Cultivated populations***	8 (7)^3^	
***Total***	**130**	**216**

Populations from the SARDI (AMGRC) collection were represented by a single individual.

^1 ^among these 26 populations, 14 were collected in Corsica.

^2 ^among these 15 populations 4 were collected in Crete.

^3 ^the cultivated populations are: Borung, Caliph, Cyprus, Jemalong, Mogul, Paraggio and Sephi (we used 1 line per population except for Cyprus which was represented by 2 lines).

**Table 2:** Primers and characteristics of microsatellites loci.

Locus	Motif	Origin	Function	Primer sequences (5'-3')	Temp	Linkage group	Genebank Accession
FMT 07	[GA]_28_	GL	Non coding	GCAGAGAAAAGTAACACCATC CAGGTTCTTTCTTACTTTCCC	60	7	AY294632
FMT 08	[CT]_16 _[CA]_7_	GL	Non coding	CAATCACTGGAAGCAAGGT AGCCTGCTCATTTGTATTGC	55	7	AY294633
FMT 11	[GA]_16_	GL	Non coding	GGCCCAACCACAATTTC CATAACTTCCAATAACTGCCA	55	1	AY294635
FMT 50	[GA]_10_	GL	Non coding	TAGGGACTTGAGGGGGTG CAATATCGTAACACATGGCTC	60	3	AY294640
FMT 90	[GA]_12_	GL	Non coding	GGATGATGAAGACAATGTTAGCA AGAAGTTATAGAGGGACGAAATTAG	60	1	AY294642
FMTBN1	[TA]_6_	GB	Non coding	TGTCAATTTCAGTGTTTGCT AGTAAATGACAATAAGGAGT	50	5	AC126010
MAL367466	[TTC]_8_	GB	Unknown	ACACAAACAAACATTCTCAC CATCGGTTGTTAGAGAGAG	60	2	AL367466
ENPB1	[AC]_6_	GB	ENBP 1 gene	CACTTCCCACTGTCCTAGTCCTAC GACTCGTCATCACCAGTTTCATCC	55	1	AJ002479
MTIC37	[AAG]_10_	GB	Unknown	AAGAGCAAGCAAGAGGATGC TCTTGGCCATTACAATATCATCA	55	4	AL373844
MTIC59	[AAG]_6_	GB	Unknown	CAAATTTGGAGAAGGCGAAG CGGTCTCTTCATATTCGCTGA	55	5	AL377524
MTIC126	[AC]_8_	GB	Unknown	CCCAGTTGCAGACTCTCTCA GAAGGGTTTACCGGAGGAAG	50	6	AL368250
MTIC243	[TTG]_6_	GB	Unknown	GGAGGAGGTTATAGGTTTGG TCAGTGCTCAGCATCTATGT	50	6	n.a.
MTPG85C	[TC]_11 _[AC]_8_	GL	Non coding	CCAACACAGTCACCTAACCAA GTTTCTCCATTGTTTCGTAGG	50	?	AF274878

Microsatellite motif was determined based on the sequence of a cloned allele. Microsatellite loci were obtained from microsatellite-enriched genomic libraries (GL) or using sequence information retrieved from GenBank (GB).

Temp: optimal annealing temperature for the PCR amplification

n.a. non available

**Table 3:** Summary of microsatellite diversity detected in the collection.

Microsatellite marker	*N*a	*N*a, p	*H*_E_	*H*_O_
FMT11	18	11	0.925	0.036
FMT90	22	8	0.916	0.020
ENPB1	20	8	0.877	0.043
FMT50	29	5	0.866	0.042
MTIC37	16	4	0.825	0.045
MTIC59	26	7	0.893	0.047
FMTBN1	53	5	0.964	0.020
MTIC243	5	2	0.191	0.009
MTIC126	3	1	0.073	0.003
FMT08C	23	7	0.911	0.023
FMT07	31	8	0.928	0.012
MTPG85C	16	7	0.853	0.006
MAL367466	7	3	0.489	0.017
Mean (SD)	20.7	5.8	0.747 (0.298)	0.025 (0.016)

Number of allele per locus (*N*a), number of alleles with a frequency higher than 5% (*N*a, p), expected (*H*_E_) and observed (*H*_O_) heterozygosity revealed in the whole collection (*n *= 346) for 13 microsatellite loci.

**Table 4:** Allelic diversity and proportion of loci in linkage disequilibrium in the four clusters inferred using Structure (*n *= 346).

**Cluster**	**Number of individuals**	***N***_**A**_	***H*_**E **_(SE)**	**Number of pairs of loci in LD (# of comparisons)**
Whole sample	346	20.7	0.747 (0.300)	29 (78)
				
1	83	12.8	0.699 (0.277)	4 (77)
2	99	13.9	0.696 (0.287)	4 (78)
3	114	13.1	0.680 (0.308)	1 (78)
4	50	11.0	0.741 (0.291)	5 (75)

Clusters 1 to 4 refer respectively to red, blue, yellow and green symbols in Figure 1.

Pairs of markers where considered in significant LD using the threshold α = 0.05 after using a sequential Bonferroni correction

**Table 5:** Between cluster differentiation (*F*_ST_) obtained at *K *= 4 (*n *= 346).

	Cluster 2	Cluster 3	Cluster 4
Cluster 1	0.094	0.054	0.083
Cluster 2		0.094	0.102
Cluster 3			0.077

All values are highly significant (*p *< 0.001).
