# Supplementary material for: Microsatellite diversity and broad scale geographic structure in a model legume: building a set of nested core collection for studying naturally occurring variation in Medicago truncatula
Source: BMC Plant Biol. 2006 Dec 13;6:28. doi: 10.1186/1471-2229-6-28 (PMC1762007; doi:10.1186/1471-2229-6-28)
Supplement: Additional File 2 — Figure S1 (Log) Likelihood of the data (n = 266) as a function of K (the number of groups used to stratify the sample). For each K value, 5 independent runs were considered. [file 1471-2229-6-28-S2.pdf]

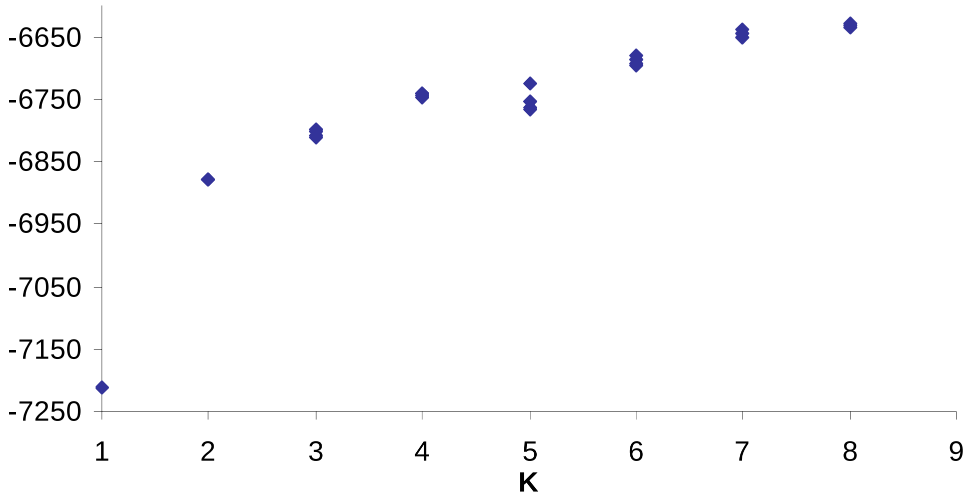

Figure S1: (Log) Likelihood of the data (*n* = 266) as a function of *K* (the number of groups used to stratify the sample). For each *K* value, 5 independent runs (blue diamonds) were considered.
