# Supplementary material for: Microsatellite diversity and broad scale geographic structure in a model legume: building a set of nested core collection for studying naturally occurring variation in Medicago truncatula
Source: BMC Plant Biol. 2006 Dec 13;6:28. doi: 10.1186/1471-2229-6-28 (PMC1762007; doi:10.1186/1471-2229-6-28)
Supplement: Additional File 3 — Table S2: Composition of groups obtained for different values of K (n = 266). [file 1471-2229-6-28-S3.pdf]

Table S2: Composition of clusters obtained for different values of K ($n = 266$).

	cluster composition								Partial membership
**K=2**	0.87<br>(178)<br>5	0.86<br>(88)<br>9							14
**K=3**	0.65<br>(93)<br>37	0.77<br>(78)<br>14	0.70<br>(95)<br>36						87
**K=4**	0.70<br>(71)<br>24	0.72<br>(68)<br>17	0.63<br>(85)<br>36	0.66<br>(42)<br>15					92
**K=5**	0.63<br>(72)<br>18	0.64<br>(27)<br>28	0.68<br>(61)<br>12	0.59<br>(37)<br>38	0.59<br>(69)<br>21				117
**K=6**	0.57<br>(56)<br>37	0.65<br>(60)<br>23	0.55<br>(31)<br>17	0.63<br>(24)<br>10	0.60<br>(33)<br>17	0.53<br>(62)<br>44			148
**K=7**	0.53<br>(57)<br>10	0.54<br>(64)<br>21	0.65<br>(21)<br>8	0.62<br>(23)<br>39	0.54<br>(52)<br>17	0.50<br>(27)<br>36	0.58<br>(32)<br>21		152
**K=8**	0.59<br>(24)<br>11	0.58<br>(31)<br>17	0.66<br>(19)<br>7	0.52<br>(50)<br>33	0.34<br>(43)<br>43	0.34<br>(18)<br>18	0.51<br>(27)<br>15	0.62<br>(54)<br>22	166

Notes: In each table entry, the mean proportion of membership in each cluster, followed by the number of individuals assigned to each cluster (between parentheses) and the number of individuals showing a partial membership in this group ($p < 0.6$) is given.
The last column "partial membership" gives the total number of individuals with partial membership ($p < 0.6$). For each K value, the results presented correspond to the run yielding the highest posterior probability of the data.
