# Supplementary material for: Microsatellite diversity and broad scale geographic structure in a model legume: building a set of nested core collection for studying naturally occurring variation in Medicago truncatula
Source: BMC Plant Biol. 2006 Dec 13;6:28. doi: 10.1186/1471-2229-6-28 (PMC1762007; doi:10.1186/1471-2229-6-28)
Supplement: Additional File 4 — Table S3 Composition of the set of nested core-collections assembled to represent naturally occurring variation in Medicago truncatula ssp truncatula. [file 1471-2229-6-28-S4.doc]

Table S3. Composition of the set of nested core-collections assembled to represent naturally occurring variation in *Medicago* *truncatula* ssp *truncatula.*

| Core collection | Line number | Population^1^ | Country^2^ | Latitude | Longitude | Cluster^3^ | Max p^4^ |
| --- | --- | --- | --- | --- | --- | --- | --- |
| CC8 | 163 | SA.22322 | Syria | 35.017 | -37.100 | 1 | 0.52 |
| CC8 | 174 | SA.28064 | Cyprus | 34.783 | -33.167 | 1 | 0.88 |
| CC8 | 544 | ESP105 | Spain | 38.076 | 3.816 | 2 | 0.91 |
| CC8 | 736 | DZA045 | Algeria | 36.923 | -7.736 | 3 | 0.36 |
| CC8 | 734 | DZA315 | Algeria | 34.716 | -0.158 | 3 | 0.68 |
| CC8 | 530 | F83005 | France | 43.330 | -6.140 | 3 | 0.55 |
| CC8 | 651 | F66014 | France | 42.490 | -2.560 | 4 | 0.43 |
| CC8 | 368 | DZA012 | Algeria | 36.549 | -3.183 | 4 | 0.85 |
| CC16 | 555 | GRC020 | Greece | 38.122 | -21.543 | 1 | 0.90 |
| CC16 | 154 | SA.24714 | Italy | 37.533 | -14.517 | 1 | 0.70 |
| CC16 | 543 | DZA327 | Algeria | 35.252 | 0.703 | 2 | 0.42 |
| CC16 | 239 | SA.26063 | Morocco | 32.167 | 8.833 | 2 | 0.83 |
| CC16 | 648 | F66014 | France | 42.490 | -2.560 | 2 | 0.60 |
| CC16 | 542 | DZA233 | Algeria | 35.847 | -4.951 | 3 | 0.87 |
| CC16 | 550 | F11013 | France | 43.090 | -2.520 | 4 | 0.84 |
| CC16 | 49 | SA.09707 | Tunisia | 36.000 | -8.000 | 4 | 0.63 |
| CC32 | 552 | F20047 | France | 42.582 | -9.060 | 1 | 0.49 |
| CC32 | 337 | GRC042 | Greece | 37.641 | -23.142 | 1 | 0.90 |
| CC32 | 245 | SA.14161 | Jordan | 32.317 | -35.750 | 1 | 0.83 |
| CC32 | 321 | SA.03780 | Italy | 38.100 | -15.650 | 1 | 0.85 |
| CC32 | 545 | ESP158 | Spain | 37.529 | 5.023 | 2 | 0.88 |
| CC32 | 679 | F66017 | France | 42.370 | -2.450 | 2 | 0.48 |
| CC32 | 369 | PRT180 | Portugal | 37.193 | 8.887 | 2 | 0.62 |
| CC32 | 554 | F20089 | France | 42.971 | -9.367 | 3 | 0.76 |
| CC32 | 557 | GRC064 | Greece | 39.987 | -23.932 | 3 | 0.42 |
| CC32 | 263 | SA.03116 | Israel | 32.000 | -35.000 | 3 | 0.84 |
| CC32 | 198 | SA.09048 | Libya | 32.200 | -20.550 | 3 | 0.76 |
| CC32 | 310 | SA.09944 | Tunisia | 35.567 | -8.667 | 3 | 0.81 |
| CC32 | 144 | SA.14163 | Jordan | 32.000 | -35.000 | 3 | 0.74 |
| CC32 | 290 | SA.09119 | Turkey | 41.033 | -28.950 | 3 | 0.68 |
| CC32 | 549 | F11005 | France | 43.060 | -3.050 | 4 | 0.62 |
| CC32 | 213 | SA.27882 | Morocco | 33.083 | 6.667 | 4 | 0.71 |
| CC64 | 297 | SA.08626 | Morocco | 29.717 | 9.600 | 1 | 0.41 |
| CC64 | 228 | SA.27063 | Greece | 38.083 | -22.567 | 1 | 0.43 |
| CC64 | 61 | SA.09357 | Algeria | 36.650 | -7.417 | 1 | 0.62 |
| CC64 | 379 | GRC063 | Greece | 40.159 | -23.732 | 1 | 0.57 |
| CC64 | 654 | DZA014 | Algeria | 36.475 | -3.311 | 1 | 0.41 |
| CC64 | 234 | SA.23859 | Tunisia | 36.417 | -9.250 | 1 | 0.54 |
| CC64 | 675 | DZA326 | Algeria | 35.216 | 0.984 | 1 | 0.56 |
| CC64 | 645 | GRC033 | Greece | 36.915 | -22.500 | 1 | 0.59 |
| CC64 | 274 | SA.03648 | Portugal | 37.150 | 7.550 | 2 | 0.49 |
| CC64 | 395 | DZA309 | Algeria | 34.803 | 1.674 | 2 | 0.43 |
| CC64 | 416 | ESP074 | Spain | 41.121 | -0.988 | 2 | 0.57 |
| CC64 | 512 | ESP155 | Spain | 37.534 | 5.964 | 2 | 0.72 |
| CC64 | 357 | DZA202 | Algeria | 35.221 | -0.121 | 2 | 0.63 |
| CC64 | 639 | DZA210 | Algeria | 35.857 | -2.039 | 2 | 0.42 |
| CC64 | 360 | DZA058 | Algeria | 36.140 | -7.944 | 2 | 0.39 |
| CC64 | 276 | SA.07749 | Tunisia | 36.217 | -10.283 | 3 | 0.56 |
| CC64 | 513 | ESP163 | Spain | 36.989 | 4.285 | 3 | 0.33 |
| CC64 | 314 | SA.09866 | Algeria | 36.650 | -7.417 | 3 | 0.43 |
| CC64 | 130 | SA.12451 | Italy | 39.117 | -8.933 | 3 | 0.54 |
| CC64 | 477 | DZA058 | Algeria | 36.140 | -7.944 | 3 | 0.37 |
| CC64 | 52 | SA.09710 | Tunisia | 35.750 | -8.517 | 3 | 0.51 |
| CC64 | 475 | DZA016 | Algeria | 36.279 | -3.567 | 3 | 0.42 |
| CC64 | 371 | PRT178 | Portugal | 37.206 | 8.488 | 3 | 0.40 |
| CC64 | 525 | PRT176 | Portugal | 37.153 | 7.726 | 3 | 0.43 |
| CC64 | 204 | SA.01489 | Israel | 32.683 | -35.400 | 3 | 0.45 |
| CC64 | 232 | SA.27062 | Greece | 38.083 | -22.567 | 3 | 0.49 |
| CC64 | 307 | SA.08625 | Morocco | 33.000 | 6.000 | 4 | 0.75 |
| CC64 | 370 | PRT179 | Portugal | 37.075 | 8.800 | 4 | 0.42 |
| CC64 | 265 | SA.01526 | Algeria | 36.000 | -3.000 | 4 | 0.70 |
| CC64 | 520 | F20015 | France | 42.084 | -9.369 | 4 | 0.51 |
| CC64 | 601 | DZA246 | Algeria | 36.302 | -7.150 | 4 | 0.55 |
| CC64 | 449 | F11012 | France | 43.000 | -2.440 | 4 | 0.86 |
| CC96 | 126 | SA.11959 | Israel | 33.000 | -35.000 | 1 | 0.51 |
| CC96 | 637 | DZA061 | Algeria | 36.500 | -7.404 | 1 | 0.51 |
| CC96 | 356 | DZA202 | Algeria | 35.221 | -0.121 | 1 | 0.53 |
| CC96 | 178 | SA.28097 | Cyprus | 34.000 | -33.000 | 1 | 0.89 |
| CC96 | 225 | SA.21560 | Libya | 31.417 | -15.483 | 1 | 0.80 |
| CC96 | 362 | DZA045 | Algeria | 36.923 | -7.736 | 1 | 0.43 |
| CC96 | 458 | F20025 | France | 42.756 | -9.451 | 1 | 0.33 |
| CC96 | 202 | SA.02840 | Cyprus | 35.000 | -33.000 | 1 | 0.81 |
| CC96 | 397 | DZA323 | Algeria | 35.633 | 0.874 | 2 | 0.35 |
| CC96 | 444 | ESP178 | Spain | 37.391 | 6.233 | 2 | 0.87 |
| CC96 | 673 | DZA319 | Algeria | 35.288 | 0.571 | 2 | 0.80 |
| CC96 | 443 | ESP174 | Spain | 37.333 | 6.761 | 2 | 0.43 |
| CC96 | 497 | DZA323 | Algeria | 35.633 | 0.874 | 2 | 0.76 |
| CC96 | 431 | ESP155 | Spain | 37.534 | 5.964 | 2 | 0.37 |
| CC96 | 437 | ESP163 | Spain | 36.989 | 4.285 | 2 | 0.79 |
| CC96 | 548 | ESP171 | Spain | 36.388 | 6.113 | 3 | 0.35 |
| CC96 | 394 | DZA243 | Algeria | 35.809 | -7.367 | 3 | 0.71 |
| CC96 | 47 | SA.09670 | Algeria | 34.833 | 1.350 | 3 | 0.41 |
| CC96 | 60 | SA.09434 | Tunisia | 35.833 | -9.200 | 3 | 0.62 |
| CC96 | 293 | SA.09137 | Algeria | 34.833 | 0.167 | 3 | 0.43 |
| CC96 | 522 | F83005 | France | 43.330 | -6.140 | 3 | 0.44 |
| CC96 | 317 | SA.09715 | Tunisia | 35.750 | -8.517 | 3 | 0.65 |
| CC96 | 523 | PRT176 | Portugal | 37.153 | 7.726 | 3 | 0.35 |
| CC96 | 219 | SA.27192 | Italy | 41.300 | -17.783 | 3 | 0.62 |
| CC96 | 674 | DZA322 | Algeria | 35.387 | 0.841 | 3 | 0.37 |
| CC96 | 460 | F20026 | France | 42.924 | -9.357 | 3 | 0.56 |
| CC96 | 450 | F11008 | France | 42.550 | -2.560 | 4 | 0.74 |
| CC96 | 574 | F13006 | France | 43.250 | -5.120 | 4 | 0.41 |
| CC96 | 303 | SA.08604 | Algeria | 36.250 | -2.800 | 4 | 0.49 |
| CC96 | 354 | DZA210 | Algeria | 35.857 | -2.039 | 4 | 0.61 |
| CC96 | 355 | DZA210 | Algeria | 35.857 | -2.039 | 4 | 0.43 |
| CC96 | 302 | SA.04087 | Tunisia | 36.950 | -10.217 | 4 | 0.63 |

Line number refers to Table S1 [see Additional file 1].

(1). Original accession name or population from which the inbred line has been extracted.

(2). Country in which the population/accession has been initially collected.

(3). Cluster to which the inbred line was assigned when stratifying the collection in *K* = 4 groups (see text for details on the inference of stratification and the procedure used to assign inbred line to each group).

(4) Proportion of membership in the group. Values close to 1 denote accessions assigned with high probability to a single group whereas intermediate values (0.3 - 0.5) denote accessions that are intermediate between two groups or sometimes three groups.
