# Supplementary material for: Microsatellite diversity and broad scale geographic structure in a model legume: building a set of nested core collection for studying naturally occurring variation in Medicago truncatula
Source: BMC Plant Biol. 2006 Dec 13;6:28. doi: 10.1186/1471-2229-6-28 (PMC1762007; doi:10.1186/1471-2229-6-28)
Supplement: Additional File 1 — Table S1 list of the 346 accessions (inbred lines) analysed in the present study and associated geographical data. [file 1471-2229-6-28-S1.pdf]

Table S1

Sample number	Accession number	Original population name	Stock center	Origin	Latitude	Longitude
1	L00043	SA.09356	SARDI	Algeria	36.650	-7.417
2	L00044	SA.09456	SARDI	Tunisia	34.750	-10.717
3	L00045	SA.09596	SARDI	Tunisia	35.083	-8.667
4	L00046	SA.09642	SARDI	Algeria	36.183	-5.467
5	L00047	SA.09670	SARDI	Algeria	34.833	1.350
6	L00048	SA.09693	SARDI	Tunisia	36.000	-9.333
7	L00049	SA.09707	SARDI	Tunisia	36.000	-8.000
8	L00052	SA.09710	SARDI	Tunisia	35.750	-8.517
9	L00053	SA.09712	SARDI	Tunisia	35.750	-8.517
10	L00054	SA.19995	SARDI	Cyprus	34.000	-32.000
11	L00057	SA.09700	SARDI	Tunisia	36.350	-9.617
12	L00059	SA.09649	SARDI	Algeria	35.433	-6.250
13	L00060	SA.09434	SARDI	Tunisia	35.833	-9.200
14	L00061	SA.09357	SARDI	Algeria	36.650	-7.417
15	L00126	SA.11959	SARDI	Israel	33.000	-35.000
16	L00130	SA.12451	SARDI	Italy	39.117	-8.933
17	L00132	SA.10481	SARDI	Tunisia	36.833	-10.217
18	L00134	SA.19998	SARDI	Cyprus	34.733	-33.167
19	L00139	SA.15268	SARDI	Algeria	35.000	0.650
20	L00144	SA.14163	SARDI	Jordan	32.000	-35.000
21	L00145	SA.21932	SARDI	Algeria	36.300	-6.550
22	L00146	SA.19964	SARDI	Turkey	37.883	-28.500
23	L00147	SA.19983	SARDI	Cyprus	34.667	-33.083
24	L00148	SA.21819	SARDI	Cyprus	34.700	-33.050
25	L00153	SA.17562	SARDI	Greece	35.167	-25.033
26	L00154	SA.24714	SARDI	Italy	37.533	-14.517
27	L00157	SA.27138	SARDI	Greece	37.933	-22.917
28	L00161	SA.21362	SARDI	Libya	31.417	-15.250
29	L00162	SA.21590	SARDI	Libya	31.250	-16.033
30	L00163	SA.22322	SARDI	Syria	35.017	-37.100
31	L00165	SA.25226	SARDI	Italy	37.133	-14.917
32	L00166	SA.25654	SARDI	Morocco	33.883	5.617
33	L00167	SA.27176	SARDI	Greece	38.483	-22.500
34	L00168	SA.25898	SARDI	Italy	39.133	-9.533
35	L00170	SA.27774	SARDI	Morocco	33.083	7.617
36	L00172	SA.27185	SARDI	Italy	41.300	-17.783
37	L00173	SA.27778	SARDI	Morocco	32.550	7.683
38	L00174	SA.28064	SARDI	Cyprus	34.783	-33.167
39	L00177	SA.28095	SARDI	Cyprus	34.650	-33.767
40	L00178	SA.28097	SARDI	Cyprus	34.000	-33.000
41	L00179	SA.28889	SARDI	France	41.600	-8.083
42	L00180	SA.28890	SARDI	France	41.717	-8.783
43	L00181	SA.28339	SARDI	Spain	37.067	5.083
44	L00188	SA.31442	SARDI	Morocco	33.533	6.267
45	L00192	SA.30740	SARDI	Romania	44.467	-26.500
46	L00195	SA.30199	SARDI	South Africa	-34.083	-20.917

47	L00197	SA.30203	SARDI	South Africa	-34.000	-20.000
48	L00198	SA.09048	SARDI	Libya	32.200	-20.550
49	L00201	SA.03047	SARDI	Portugal	38.867	7.283
50	L00202	SA.02840	SARDI	Cyprus	35.000	-33.000
51	L00203	SA.02831	SARDI	Spain	38.100	3.767
52	L00204	SA.01489	SARDI	Israel	32.683	-35.400
53	L00207	SA.09970	SARDI	Tunisia	35.083	-8.667
54	L00211	SA.31443	SARDI	Morocco	33.083	7.583
55	L00212	SA.28375	SARDI	Portugal	37.067	8.800
56	L00213	SA.27882	SARDI	Morocco	33.083	6.667
57	L00214	SA.28110	SARDI	Cyprus	34.950	-34.083
58	L00215	SA.28099	SARDI	Cyprus	34.000	-33.000
59	L00216	SA.27961	SARDI	Morocco	31.833	6.933
60	L00217	SA.28089	SARDI	Cyprus	34.900	-33.617
61	L00218	SA.27942	SARDI	Morocco	33.650	7.167
62	L00219	SA.27192	SARDI	Italy	41.300	-17.783
63	L00221	SA.25926	SARDI	Italy	39.100	-9.533
64	L00222	SA.25915	SARDI	Italy	39.000	-9.000
65	L00223	SA.25664	SARDI	Morocco	34.000	6.000
66	L00224	SA.27159	SARDI	Greece	37.933	-22.917
67	L00225	SA.21560	SARDI	Libya	31.417	-15.483
68	L00226	SA.25941	SARDI	Italy	39.000	-9.000
69	L00228	SA.27063	SARDI	Greece	38.083	-22.567
70	L00229	SA.27129	SARDI	Greece	37.933	-22.917
71	L00231	SA.27137	SARDI	Greece	37.933	-22.917
72	L00232	SA.27062	SARDI	Greece	38.083	-22.567
73	L00233	SA.24576	SARDI	Morocco	34.083	5.000
74	L00234	SA.23859	SARDI	Tunisia	36.417	-9.250
75	L00237	SA.21302	SARDI	Libya	32.667	-14.250
76	L00238	SA.22323	SARDI	Syria	35.017	-37.083
77	L00239	SA.26063	SARDI	Morocco	32.167	8.833
78	L00241	SA.15951	SARDI	Madeira	32.667	16.917
79	L00242	SA.14829	SARDI	Algeria	35.000	0.650
80	L00244	SA.18543	SARDI	Tunisia	35.567	-8.667
81	L00245	SA.14161	SARDI	Jordan	32.317	-35.750
82	L00246	SA.12455	SARDI	Italy	39.000	-8.000
83	L00247	SA.18532	SARDI	Tunisia	35.833	-9.167
84	L00248	SA.10406	SARDI	Morocco	33.000	6.000
85	L00258	SA.02841	SARDI	Cyprus	35.000	-33.000
86	L00263	SA.03116	SARDI	Israel	32.000	-35.000
87	L00265	SA.01526	SARDI	Algeria	36.000	-3.000
88	L00267	SA.02084	SARDI	Greece	37.550	-25.133
89	L00270	SA.02820	SARDI	Turkey	36.967	-30.800
90	L00274	SA.03648	SARDI	Portugal	37.150	7.550
91	L00275	SA.04947	SARDI	Australia	-35.150	-141.983
92	L00276	SA.07749	SARDI	Tunisia	36.217	-10.283
93	L00277	SA.03653	SARDI	Portugal	38.733	9.133
94	L00280	SA.07763	SARDI	Tunisia	36.217	-10.283
95	L00281	SA.03562	SARDI	France	43.700	-7.267
96	L00283	SA.08454	SARDI	Libya	32.500	-20.833
97	L00285	SA.08916	SARDI	Algeria	36.183	-5.400
98	L00286	SA.09049	SARDI	Libya	32.483	-20.833

99	L00287	SA.09062	SARDI	Australia	-33.000	-134.250
100	L00288	SA.09851	SARDI	Algeria	36.250	-2.283
101	L00289	SA.09856	SARDI	Algeria	35.367	-7.150
102	L00290	SA.09119	SARDI	Turkey	41.033	-28.950
103	L00292	SA.09138	SARDI	Libya	32.500	-20.833
104	L00293	SA.09137	SARDI	Algeria	34.833	0.167
105	L00296	SA.08642	SARDI	Morocco	35.667	5.867
106	L00297	SA.08626	SARDI	Morocco	29.717	9.600
107	L00298	SA.08618	SARDI	Morocco	33.783	6.833
108	L00299	SA.08619	SARDI	Morocco	33.667	6.667
109	L00301	SA.08601	SARDI	Algeria	36.183	-5.400
110	L00302	SA.04087	SARDI	Tunisia	36.950	-10.217
111	L00303	SA.08604	SARDI	Algeria	36.250	-2.800
112	L00305	SA.08603	SARDI	Algeria	36.183	-5.400
113	L00306	SA.08623	SARDI	Morocco	33.067	7.000
114	L00307	SA.08625	SARDI	Morocco	33.000	6.000
115	L00308	SA.09888	SARDI	Morocco	33.667	6.667
116	L00309	SA.08638	SARDI	Morocco	33.167	5.633
117	L00310	SA.09944	SARDI	Tunisia	35.567	-8.667
118	L00311	SA.09876	SARDI	Algeria	36.217	-2.767
119	L00313	SA.09728	SARDI	Tunisia	34.750	-10.717
120	L00314	SA.09866	SARDI	Algeria	36.650	-7.417
121	L00315	SA.09820	SARDI	Libya	32.000	-20.000
122	L00316	SA.09720	SARDI	Tunisia	35.567	-8.667
123	L00317	SA.09715	SARDI	Tunisia	35.750	-8.517
124	L00321	SA.03780	SARDI	Italy	38.100	-15.650
125	L00322	SA.03749	SARDI	Israel	32.000	-35.000
126	L00324	SA.04586	SARDI	Tunisia	36.000	-9.000
127	L00325	SA.06088	SARDI	Portugal	38.733	9.133
128	L00330	SA.02806	SARDI	Portugal	38.867	7.283
129	L00332	SA.02748	SARDI	Israel	32.800	-35.533
130	L00334	SA.03308	SARDI	Australia	-33.633	-137.917
131	L00337	GRC042	INRA-Montpellier	Greece	37.641	-23.142
132	L00338	CRE009	INRA-Montpellier	Greece	35.035	-25.935
133	L00339	CRE006	INRA-Montpellier	Greece	35.062	-24.962
134	L00340	CRE005	INRA-Montpellier	Greece	35.115	-24.688
135	L00341	DZA242	INRA-Montpellier	Algeria	35.509	-6.892
136	L00342	DZA241	INRA-Montpellier	Algeria	35.487	-6.478
137	L00343	DZA236	INRA-Montpellier	Algeria	36.014	-6.567
138	L00344	DZA231	INRA-Montpellier	Algeria	36.108	-4.944
139	L00345	DZA230	INRA-Montpellier	Algeria	36.191	-5.383
140	L00346	DZA230	INRA-Montpellier	Algeria	36.191	-5.383
141	L00348	DZA202	INRA-Montpellier	Algeria	35.221	-0.121
142	L00350	DZA215	INRA-	Algeria	34.916	-1.527

			Montpellier			
143	L00351	DZA213	INRA-Montpellier	Algeria	35.320	-1.500
144	L00352	DZA212	INRA-Montpellier	Algeria	35.392	-1.348
145	L00353	DZA212	INRA-Montpellier	Algeria	35.392	-1.348
146	L00354	DZA210	INRA-Montpellier	Algeria	35.857	-2.039
147	L00355	DZA210	INRA-Montpellier	Algeria	35.857	-2.039
148	L00356	DZA202	INRA-Montpellier	Algeria	35.221	-0.121
149	L00357	DZA202	INRA-Montpellier	Algeria	35.221	-0.121
150	L00358	DZA061	INRA-Montpellier	Algeria	36.500	-7.404
151	L00359	DZA059	INRA-Montpellier	Algeria	36.203	-7.939
152	L00360	DZA058	INRA-Montpellier	Algeria	36.140	-7.944
153	L00361	DZA046	INRA-Montpellier	Algeria	36.977	-7.551
154	L00362	DZA045	INRA-Montpellier	Algeria	36.923	-7.736
155	L00363	DZA045	INRA-Montpellier	Algeria	36.923	-7.736
156	L00364	DZA033	INRA-Montpellier	Algeria	36.509	-5.394
157	L00365	DZA033	INRA-Montpellier	Algeria	36.509	-5.394
158	L00366	DZA022	INRA-Montpellier	Algeria	36.355	-5.428
159	L00367	DZA016	INRA-Montpellier	Algeria	36.279	-3.567
160	L00369	PRT180	INRA-Montpellier	Portugal	37.193	8.887
161	L00370	PRT179	INRA-Montpellier	Portugal	37.075	8.800
162	L00371	PRT178	INRA-Montpellier	Portugal	37.206	8.488
163	L00372	PRT177	INRA-Montpellier	Portugal	37.190	8.081
164	L00373	PRT176	INRA-Montpellier	Portugal	37.153	7.726
165	L00374	PRT176	INRA-Montpellier	Portugal	37.153	7.726
166	L00375	GRC098	INRA-Montpellier	Greece	38.203	-22.138
167	L00376	GRC093	INRA-Montpellier	Greece	38.347	-22.356
168	L00379	GRC063	INRA-Montpellier	Greece	40.159	-23.732
169	L00380	GRC063	INRA-Montpellier	Greece	40.159	-23.732
170	L00381	GRC052	INRA-Montpellier	Greece	39.242	-23.228
171	L00383	GRC040	INRA-Montpellier	Greece	37.596	-23.011

172	L00384	GRC037	INRA-Montpellier	Greece	37.023	-22.534
173	L00385	GRC033	INRA-Montpellier	Greece	36.915	-22.500
174	L00386	GRC033	INRA-Montpellier	Greece	36.915	-22.500
175	L00387	GRC024	INRA-Montpellier	Greece	37.723	-21.874
176	L00388	GRC024	INRA-Montpellier	Greece	37.723	-21.874
177	L00391	DZA243	INRA-Montpellier	Algeria	35.809	-7.367
178	L00392	DZA242	INRA-Montpellier	Algeria	35.509	-6.892
179	L00393	DZA244	INRA-Montpellier	Algeria	35.905	-7.178
180	L00394	DZA243	INRA-Montpellier	Algeria	35.809	-7.367
181	L00395	DZA309	INRA-Montpellier	Algeria	34.803	1.674
182	L00396	DZA246	INRA-Montpellier	Algeria	36.302	-7.150
183	L00397	DZA323	INRA-Montpellier	Algeria	35.633	0.874
184	L00398	DZA312	INRA-Montpellier	Algeria	35.194	0.352
185	L00400	DZA323	INRA-Montpellier	Algeria	35.633	0.874
186	L00401	ESP031	INRA-Montpellier	Spain	37.500	1.991
187	L00403	ESP040	INRA-Montpellier	Spain	36.737	4.333
188	L00404	ESP039	INRA-Montpellier	Spain	36.950	4.122
189	L00405	ESP041	INRA-Montpellier	Spain	36.636	4.739
190	L00406	ESP040	INRA-Montpellier	Spain	36.737	4.333
191	L00407	ESP042	INRA-Montpellier	Spain	36.577	4.843
192	L00408	ESP041	INRA-Montpellier	Spain	36.636	4.739
193	L00409	ESP045	INRA-Montpellier	Spain	36.449	5.442
194	L00410	ESP043	INRA-Montpellier	Spain	36.420	5.125
195	L00411	ESP048	INRA-Montpellier	Spain	36.429	5.703
196	L00412	ESP048	INRA-Montpellier	Spain	36.429	5.703
197	L00414	ESP050	INRA-Montpellier	Spain	37.060	5.808
198	L00416	ESP074	INRA-Montpellier	Spain	41.121	-0.988
199	L00417	ESP095	INRA-Montpellier	Spain	36.986	2.914
200	L00421	ESP098	INRA-Montpellier	Spain	37.104	4.232
201	L00422	ESP096	INRA-	Spain	36.982	3.142

			Montpellier			
202	L00423	ESP099	INRA-Montpellier	Spain	36.953	4.342
203	L00424	ESP098	INRA-Montpellier	Spain	37.082	4.236
204	L00425	ESP100	INRA-Montpellier	Spain	37.060	4.626
205	L00426	ESP100	INRA-Montpellier	Spain	37.060	4.626
206	L00427	ESP103	INRA-Montpellier	Spain	37.728	4.854
207	L00428	ESP101	INRA-Montpellier	Spain	37.083	5.096
208	L00429	ESP104	INRA-Montpellier	Spain	37.946	4.525
209	L00430	ESP104	INRA-Montpellier	Spain	37.946	4.525
210	L00431	ESP155	INRA-Montpellier	Spain	37.534	5.964
211	L00432	ESP140	INRA-Montpellier	Spain	42.612	1.816
212	L00434	ESP156	INRA-Montpellier	Spain	37.641	5.748
213	L00435	ESP161	INRA-Montpellier	Spain	37.348	4.384
214	L00436	ESP160	INRA-Montpellier	Spain	37.418	4.419
215	L00437	ESP163	INRA-Montpellier	Spain	36.989	4.285
216	L00438	ESP162	INRA-Montpellier	Spain	37.245	4.315
217	L00439	ESP169	INRA-Montpellier	Spain	36.075	5.668
218	L00440	ESP163	INRA-Montpellier	Spain	36.989	4.285
219	L00441	ESP172	INRA-Montpellier	Spain	36.819	6.059
220	L00442	ESP170	INRA-Montpellier	Spain	36.261	5.946
221	L00443	ESP174	INRA-Montpellier	Spain	37.333	6.761
222	L00444	ESP178	INRA-Montpellier	Spain	37.391	6.233
223	L00445	ESP175	INRA-Montpellier	Spain	37.284	7.122
224	L00446	ESP174	INRA-Montpellier	Spain	37.333	6.761
225	L00447	F11007	INRA-Montpellier	France	42.590	-2.570
226	L00448	ESP175	INRA-Montpellier	Spain	37.284	7.122
227	L00449	F11012	INRA-Montpellier	France	43.000	-2.440
228	L00450	F11008	INRA-Montpellier	France	42.550	-2.560
229	L00451	F11013	INRA-Montpellier	France	43.090	-2.520
230	L00454	F20009	INRA-Montpellier	France	42.940	-9.457

231	L00455	F20025	INRA-Montpellier	France	42.756	-9.451
232	L00456	F20015	INRA-Montpellier	France	42.084	-9.369
233	L00457	F20026	INRA-Montpellier	France	42.924	-9.357
234	L00458	F20025	INRA-Montpellier	France	42.756	-9.451
235	L00460	F20026	INRA-Montpellier	France	42.924	-9.357
236	L00461	F20031	INRA-Montpellier	France	42.182	-9.377
237	L00463	F20058	INRA-Montpellier	France	41.405	-9.127
238	L00464	F20048	INRA-Montpellier	France	42.283	-9.521
239	L00465	F20061	INRA-Montpellier	France	42.183	-9.395
240	L00467	F20069	INRA-Montpellier	France	42.592	-8.908
241	L00468	F20069	INRA-Montpellier	France	42.592	-8.908
242	L00469	F20086	INRA-Montpellier	France	42.812	-9.459
243	L00470	F20081	INRA-Montpellier	France	42.401	-9.504
244	L00471	Jemalong	SARDI	cultivar		
245	L00472	F20087	INRA-Montpellier	France	42.902	-9.470
246	L00473	Cyprus	SARDI	cultivar		
247	L00475	DZA016	INRA-Montpellier	Algeria	36.279	-3.567
248	L00476	DZA016	INRA-Montpellier	Algeria	36.279	-3.567
249	L00477	DZA058	INRA-Montpellier	Algeria	36.140	-7.944
250	L00478	DZA055	INRA-Montpellier	Algeria	36.005	-8.112
251	L00479	DZA061	INRA-Montpellier	Algeria	36.500	-7.404
252	L00480	DZA058	INRA-Montpellier	Algeria	36.140	-7.944
253	L00481	DZA213	INRA-Montpellier	Algeria	35.320	-1.500
254	L00482	ESP095	INRA-Montpellier	Spain	36.986	2.914
255	L00487	DZA230	INRA-Montpellier	Algeria	36.191	-5.383
256	L00488	DZA230	INRA-Montpellier	Algeria	36.191	-5.383
257	L00489	DZA233	INRA-Montpellier	Algeria	35.847	-4.951
258	L00490	DZA231	INRA-Montpellier	Algeria	36.108	-4.944
259	L00491	DZA241	INRA-Montpellier	Algeria	35.487	-6.478
260	L00492	DZA236	INRA-Montpellier	Algeria	36.014	-6.567
261	L00493	DZA243	INRA-	Algeria	35.809	-7.367

			Montpellier			
262	L00494	DZA241	INRA-Montpellier	Algeria	35.487	-6.478
263	L00495	DZA312	INRA-Montpellier	Algeria	35.194	0.352
264	L00496	DZA246	INRA-Montpellier	Algeria	36.302	-7.150
265	L00497	DZA323	INRA-Montpellier	Algeria	35.633	0.874
266	L00503	ESP080	INRA-Montpellier	Spain	40.441	-0.176
267	L00508	ESP095	INRA-Montpellier	Spain	36.986	2.914
268	L00509	ESP104	INRA-Montpellier	Spain	37.946	4.525
269	L00510	ESP098	INRA-Montpellier	Spain	37.104	4.232
270	L00511	ESP161	INRA-Montpellier	Spain	37.348	4.384
271	L00512	ESP155	INRA-Montpellier	Spain	37.534	5.964
272	L00513	ESP163	INRA-Montpellier	Spain	36.989	4.285
273	L00514	ESP163	INRA-Montpellier	Spain	36.989	4.285
274	L00516	ESP172	INRA-Montpellier	Spain	36.819	6.059
275	L00517	F20015	INRA-Montpellier	France	42.084	-9.369
276	L00520	F20015	INRA-Montpellier	France	42.084	-9.369
277	L00522	F83005	INRA-Montpellier	France	43.330	-6.140
278	L00523	PRT176	INRA-Montpellier	Portugal	37.153	7.726
279	L00525	PRT176	INRA-Montpellier	Portugal	37.153	7.726
280	L00526	PRT179	INRA-Montpellier	Portugal	37.075	8.800
281	L00527	Borung	SARDI	cultivar		
282	L00528	Caliph	SARDI	cultivar		
283	L00529	Cyprus	SARDI	cultivar		
284	L00530	F83005	INRA-Montpellier	France	43.330	-6.140
285	L00531	F83005	INRA-Montpellier	France	43.330	-6.140
286	L00535	Paraggio	SARDI	cultivar		
287	L00536	Sephi	SARDI	cultivar		
288	L00537	CRE007	INRA-Montpellier	Greece	35.066	-25.364
289	L00538	DZA055	INRA-Montpellier	Algeria	36.005	-8.112
290	L00539	DZA105	INRA-Montpellier	Algeria	36.883	-4.534
291	L00542	DZA233	INRA-Montpellier	Algeria	35.847	-4.951
292	L00543	DZA327	INRA-Montpellier	Algeria	35.252	0.703

293	L00544	ESP105	INRA-Montpellier	Spain	38.076	3.816
294	L00545	ESP158	INRA-Montpellier	Spain	37.529	5.023
295	L00546	ESP159	INRA-Montpellier	Spain	37.424	4.755
296	L00547	ESP165	INRA-Montpellier	Spain	36.685	4.793
297	L00548	ESP171	INRA-Montpellier	Spain	36.388	6.113
298	L00549	F11005	INRA-Montpellier	France	43.060	-3.050
299	L00550	F11013	INRA-Montpellier	France	43.090	-2.520
300	L00551	F34042	INRA-Montpellier	France	43.400	-3.460
301	L00552	F20047	INRA-Montpellier	France	42.582	-9.060
302	L00553	F20061	INRA-Montpellier	France	42.183	-9.395
303	L00554	F20089	INRA-Montpellier	France	42.971	-9.367
304	L00555	GRC020	INRA-Montpellier	Greece	38.122	-21.543
305	L00557	GRC064	INRA-Montpellier	Greece	39.987	-23.932
306	L00566	CRE007	INRA-Montpellier	Greece	35.066	-25.364
307	L00569	MOGUL	SARDI	cultivar		
308	L00573	F34050	INRA-Montpellier	France	43.300	-3.140
309	L00574	F13006	INRA-Montpellier	France	43.250	-5.120
310	L00575	DZA012	INRA-Montpellier	Algeria	36.549	-3.183
311	L00590	DZA212	INRA-Montpellier	Algeria	35.392	-1.348
312	L00598	DZA243	INRA-Montpellier	Algeria	35.809	-7.367
313	L00600	DZA244	INRA-Montpellier	Algeria	35.905	-7.178
314	L00601	DZA246	INRA-Montpellier	Algeria	36.302	-7.150
315	L00610	ESP095	INRA-Montpellier	Spain	36.986	2.914
316	L00615	ESP158	INRA-Montpellier	Spain	37.529	5.023
317	L00617	ESP175	INRA-Montpellier	Spain	37.284	7.122
318	L00620	F20058	INRA-Montpellier	France	41.405	-9.127
319	L00637	DZA061	INRA-Montpellier	Algeria	36.500	-7.404
320	L00639	DZA210	INRA-Montpellier	Algeria	35.857	-2.039
321	L00640	DZA213	INRA-Montpellier	Algeria	35.320	-1.500
322	L00642	DZA244	INRA-Montpellier	Algeria	35.905	-7.178

323	L00645	GRC033	INRA-Montpellier	Greece	36.915	-22.500
324	L00647	F66014	INRA-Montpellier	France	42.490	-2.560
325	L00648	F66014	INRA-Montpellier	France	42.490	-2.560
326	L00649	F66014	INRA-Montpellier	France	42.490	-2.560
327	L00650	F66014	INRA-Montpellier	France	42.490	-2.560
328	L00651	F66014	INRA-Montpellier	France	42.490	-2.560
329	L00652	F66014	INRA-Montpellier	France	42.490	-2.560
330	L00654	DZA014	INRA-Montpellier	Algeria	36.475	-3.311
331	L00656	DZA117	INRA-Montpellier	Algeria	37.018	-7.427
332	L00657	DZA204	INRA-Montpellier	Algeria	35.662	-0.352
333	L00659	DZA211	INRA-Montpellier	Algeria	35.536	-1.676
334	L00663	DZA226	INRA-Montpellier	Algeria	35.207	-5.703
335	L00664	DZA228	INRA-Montpellier	Algeria	35.365	-5.654
336	L00665	DZA247	INRA-Montpellier	Algeria	36.347	-7.211
337	L00666	DZA301	INRA-Montpellier	Algeria	34.995	0.739
338	L00669	DZA304	INRA-Montpellier	Algeria	34.631	0.614
339	L00673	DZA319	INRA-Montpellier	Algeria	35.288	0.571
340	L00674	DZA322	INRA-Montpellier	Algeria	35.387	0.841
341	L00675	DZA326	INRA-Montpellier	Algeria	35.216	0.984
342	L00676	DZA328	INRA-Montpellier	Algeria	35.284	0.791
343	L00679	F66017	INRA-Montpellier	France	42.370	-2.450
344	L00680	F66018	INRA-Montpellier	France	43.110	-3.000
345	L00734	DZA315	INRA-Montpellier	Algeria	34.716	-0.158
346	L00736	DZA045	INRA-Montpellier	Algeria	36.923	-7.736
